# Creation of a functional *unc-11/PICALM GFP* knock-in by CRISPR

**DOI:** 10.17912/micropub.biology.000389

**Published:** 2021-04-28

**Authors:** Maria Gallegos, Alex Hermes, Debjani Patra

**Affiliations:** 1 California State University, East Bay

## Abstract

*unc-11* is the only *C. elegans *ortholog of mammalian PICALM/AP180, paralogs that play an important role in Clathrin-Mediated Endocytosis (CME) and the recycling of a subset of SNAREs, the vesicle-associated membrane proteins (VAMPs). In this publication we report the creation of a new *unc-11* allele that is endogenously-tagged with GFP just upstream of the stop codon. Moreover, we demonstrate that the UNC-11::GFP fusion protein functions like wild type with an expression pattern similar to UNC-11 antibody staining described previously.

**Figure 1. A functional GFP knock-in at the C-terminus of UNC-11 localizes to intracellular puncta in various cell types:**
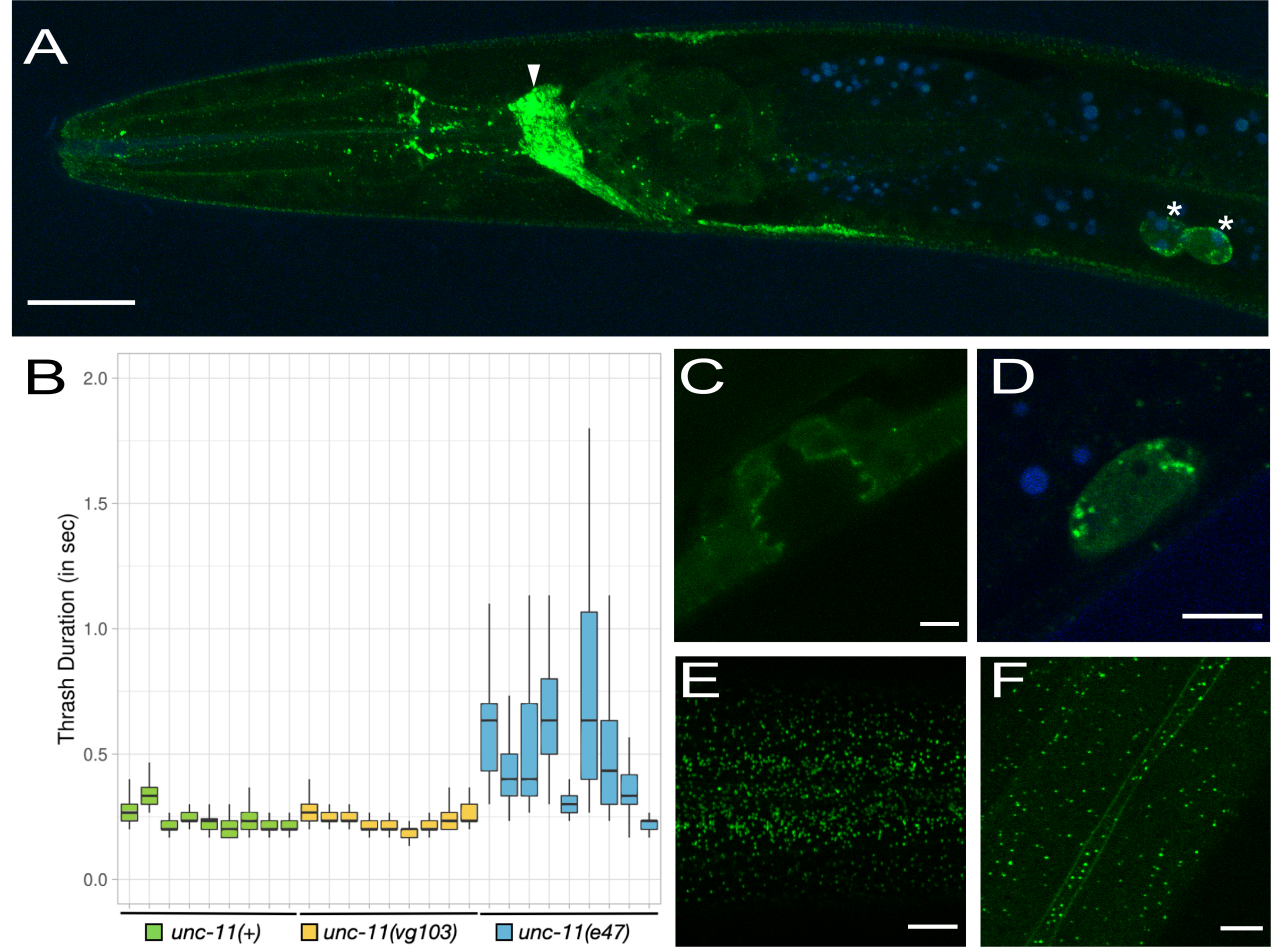
(A) A representative image of the anterior portion of an L4 *unc-11(vg103)* hermaphrodite expressing UNC-11::GFP (green) captured at 63X magnification. Blue is autofluorescence. The arrowhead points to the nerve ring and the asterisks point to coelomocytes. Scale bar indicates 20 microns. (B) Thrash duration in seconds was measured for each genotype during a 45 second interval for 9 worms each. See methods for more details. (C-F) Representative images of an L4 *unc-11(vg103)* hermaphrodite also captured at 63X magnification. Scale bar indicates 5 microns. (C) The developing vulva, (D) a coelomocyte (autofluorescence in blue), (E) the epithelial surface of the head and (F) the epithelial surface of the body centered over the seam cell membranes.

## Description

UNC-11is anortholog of mammalian PICALM (phosphatidylinositol binding clathrin assembly protein) and its neuron-specific paralog, AP180 (Maritzen *et al.* 2012). PICALM facilitates recycling of Vesicle Associated Membrane Proteins (VAMPs) from the plasma membrane (Miller *et al.* 2011, Sahlender *et al.* 2013 and Miller *et al.* 2015) and efficient clathrin-coated endocytosis (Huang *et al.* 2004, Miller *et al.* 2015 and Ishikawa *et al.* 2014). *unc-11* function in *C. elegans* is likely conservedasSNB-2 (VAMP2) and the glutamate receptor, GLR-1, both accumulate at the plasma membrane in *unc-11(e47)* null mutants (Nonet *et al.* 1999, Burbea *et al.* 2002). Mutant phenotypes outside the worm nervous system have not been described.

To determine the endogenous expression pattern and subcellular localization of *unc-11,* we created a functional GFP knock-in at the endogenous *unc-11* locus using the CRIPSR approach outlined by Dickinson *et al.* 2015. Specifically, a GFP::AID::3xFLAG coding sequence interrupted by four synthetic introns was inserted just 5′ to the *unc-11* stop codon. Successful knock-in was verified by sequencing the insertion junctions and expression of GFP.

To ask if the addition of GFP at the C-terminus of UNC-11 compromises *unc-11* function we performed a thrashing assay to examine the general health of the nervous system (Figure 1B). We compared the thrash duration (see methods) of *unc-11(+)* with the CRISPR knock-in strain, *unc-11(vg103)* and the null mutant, *unc-11(e47)*. We found no obvious differences between *unc-11(+)* and *unc-11(vg103)*. Average thrash duration during a 45 second interval was 0.251 seconds (n=9, Std. Dev. = 0.042) for *unc-11(+)* and 0.248 seconds (n=9, Std. Dev. = 0.031) for *unc-11(vg103)*. By contrast, the average thrash duration *for unc-11(e47)* was significantly higher at 0.566 seconds with a striking increase in variability (n=9, Std. Dev. = 0.196).

In general, UNC-11::GFP expression matched that of UNC-11 antibody staining described previously (Nonet *et al.* 1999) with some noted exceptions. Similar to UNC-11 antibody staining, UNC-11::GFP expression appeared pan-neuronal and was also observed within coelomocytes (Figure 1A and 1D). Not described by Nonet el al 1999, we observed GFP puncta in apical epithelial tissue just below the cuticle surface in both the head and in the body (Figure 1E and 1F), an accumulation of UNC-11::GFP at the plasma membrane in the seam cells (Figure 1F) and weak staining in the vulval precursor cells (VPCs) at the interface between the VPCs and the vulval lumen (Figure 1C). Finally, Nonet *et al.* described diffuse staining within the intestine whereas we did not observe detectable levels of intestinal expression, punctate or otherwise (Figure 1A).

Plasmid construction was done in the context of a course entitled Advanced Molecular Techniques at California State University, East Bay. This strain will be made available at the CGC. Given the inclusion of an AID tag and GFP, this strain will be useful for cell-specific protein degradation using auxin-inducible (AID) or GFP nanobody::ZIF-1 degradation methods (Wang *et al.* 2007 and Zhang *et al.* 2015).

## Methods

**Thrashing Assay:** Individual worms were placed on a glass slide with a 3% agar pad and about 75 ul of M9 buffer. Slides were then mounted onto a DIY microscope (original design by Kenji Yoshino) and videos were captured at 30 frames per second using an iPhone 7. To open the images in FIJI, the .MOV files were converted to an uncompressed AVI format using ff.WORKS software. The duration of each thrash was measured in FIJI by logging each frame number at which the worm completed a single body bend in one direction. Thrash duration (in seconds) was then calculated as the number of frames per body bend divided by 30 frames per second. Approximately, 1450 frames (~45 seconds) of video were reviewed for each animal. Body bends that did not fall below 150 degrees were not counted as a true body bend. When a body bend angle was suspected to be greater than 150 degrees, the angle ruler in Fiji (Image J) was used. Brief intervals where thrashing occurred along the Z-axis or the worm left the field of view were not included. The data was graphed using Plots of Differences available at https://huygens.science.uva.nl/PlotsOfDifferences/ (Joachim Goedhart, 2019).

**Confocal Microscopy:** *unc-11(vg103)* animals were anesthetized with sodium azide (10 mM) and imaged using a Leica SP8 confocal. To visually distinguish true GFP signal from intestinal autofluorescence for Figures 1A and 1D, Z-stacks were captured using a sequential scanning protocol: Seq 1: The 488 laser was set at 2.4%. PMT1 (green) collected wavelengths from 494 to 544 nm (capturing GFP and autofluorescence) while PMT2 (blue) collected wavelengths from 650 to 798 nm (autofluorescence only). Seq 2: The 552 laser was set at 1.0 % and PMT1 (blue) collected wavelengths from 598 to 709 nm (autofluorescence only). Adjustments were made to brightness and contrast of the blue channels to bring out the autofluorescence then the green and blue channels were combined and relevant focal planes were subject to maximum projection. The other images did not emit detectable autofluorescence and so the blue channel images were not included in the Z projection.

## Reagents

The sgRNA/Cas9 plasmid (pDQ1022) was created by Q5 SDM (NEB) using pJW1219 as template (a plasmid containing sgRNA(F+E) and Peft-3::Cas9; Addgene # 61250) and the following primer pair: CGACTAGA**CTA**TAATCCAAAgtttaagagctatgctggaa and caagacatctcgcaatagga. The *unc-11* target sequence is in all caps and the position of the *unc-11* STOP codon is in bold. The GFP::SEC repair template (pDQ1023) was created by Gibson Assembly of pJW1583 (A GFP^SEC^AID*::3xFLAG vector with ccdB sites for cloning homology arms; Addgene # 121054) digested with AvrII and SpeI and two *unc-11* homology arms (HA) amplified by PCR (Q5 DNA polymerase – NEB). Primers used to amplify the 5’HA and 3’HA homology arms from wild type genomic DNA include: ACGACGGCCAGTCGCCGGCATGCGATGGGTTAGTTTATCG and CCTGAGGCTCCCGATGCTCCTAATCCAAATGGATCGGCTG (5’HA); AGGATGACGATGACAAGAGATAGTCTAGTCGTCTATTTCTC and TATGACCATTTATCGATTTCGCAATAGAGATAAAGTTGCA (3’HA). *unc-11(vg103[unc-11::gfp])* (DQ3188) was created as described by Dickinson *et al.* 2015. Successful knock-in was verified by PCR amplification and sequencing of the junction fragments and by GFP expression.
